# Supplementary material for: End Sequence Analysis Toolkit (ESAT) expands the extractable information from single-cell RNA-seq data
Source: Genome Res. 2016 Oct;26(10):1397–410. doi: 10.1101/gr.207902.116 (PMC5052061; doi:10.1101/gr.207902.116)
Supplement: Supplemental Material [file supp_gr.207902.116_Supp_Materials.tar.gz › ESAT-esat_v0.1/doc/html/classbroad_1_1pda_1_1geneexpression_1_1agilent_1_1_make_c_l_s_from_description.html]

ESAT: broad.pda.geneexpression.agilent.MakeCLSFromDescription Class Reference


ESAT


- Main Page
- Related Pages
- Packages
- Classes
- Files

- Class List
- Class Index
- Class Hierarchy
- Class Members

All Classes Namespaces Files Functions Variables Enumerator Pages

- broad
- pda
- geneexpression
- agilent
- MakeCLSFromDescription

Public Member Functions |
Static Public Member Functions |
List of all members

broad.pda.geneexpression.agilent.MakeCLSFromDescription Class Reference

|  |  |
| --- | --- |
| Public Member Functions | |
|  | MakeCLSFromDescription (File gctFile, File descriptionFile, String save) throws IOException, ParseException |
|  | |
| void | writeCLS (String save, Map< String, ExpressionExperimentInfo > experimentInfo, File gctFile) throws IOException, ParseException |
|  | |

|  |  |
| --- | --- |
| Static Public Member Functions | |
| static void | main (String[] args) throws IOException, ParseException |
|  | |

## Constructor & Destructor Documentation

|  |  |  |  |
| --- | --- | --- | --- |
| broad.pda.geneexpression.agilent.MakeCLSFromDescription.MakeCLSFromDescription | ( | File | *gctFile*, |
|  |  | File | *descriptionFile*, |
|  |  | String | *save* |
|  | ) |  | throws IOException, ParseException |

Here is the caller graph for this function:

## Member Function Documentation

|  |  |  |  |  |  |
| --- | --- | --- | --- | --- | --- |
| static void broad.pda.geneexpression.agilent.MakeCLSFromDescription.main | ( | String[] | *args* | ) | throws IOException, ParseException |

static

Here is the call graph for this function:

|  |  |  |  |
| --- | --- | --- | --- |
| void broad.pda.geneexpression.agilent.MakeCLSFromDescription.writeCLS | ( | String | *save*, |
|  |  | Map< String, ExpressionExperimentInfo > | *experimentInfo*, |
|  |  | File | *gctFile* |
|  | ) |  | throws IOException, ParseException |

Here is the call graph for this function:

---

The documentation for this class was generated from the following file:

- java/broad/pda/geneexpression/agilent/MakeCLSFromDescription.java


---

Generated on Fri Aug 15 2014 15:11:00 for ESAT by  

 1.8.7
